# Supplementary material for: Lipid transfer proteins and PI4KIIα initiate nuclear p53-phosphoinositide signaling
Source: J Biol Chem. 2026 May 8;302(6):113123. doi: 10.1016/j.jbc.2026.113123 (PMC13260207; doi:10.1016/j.jbc.2026.113123)
Supplement: Supplementary Video 2 [file mmc2.pptx]

## Slide 1
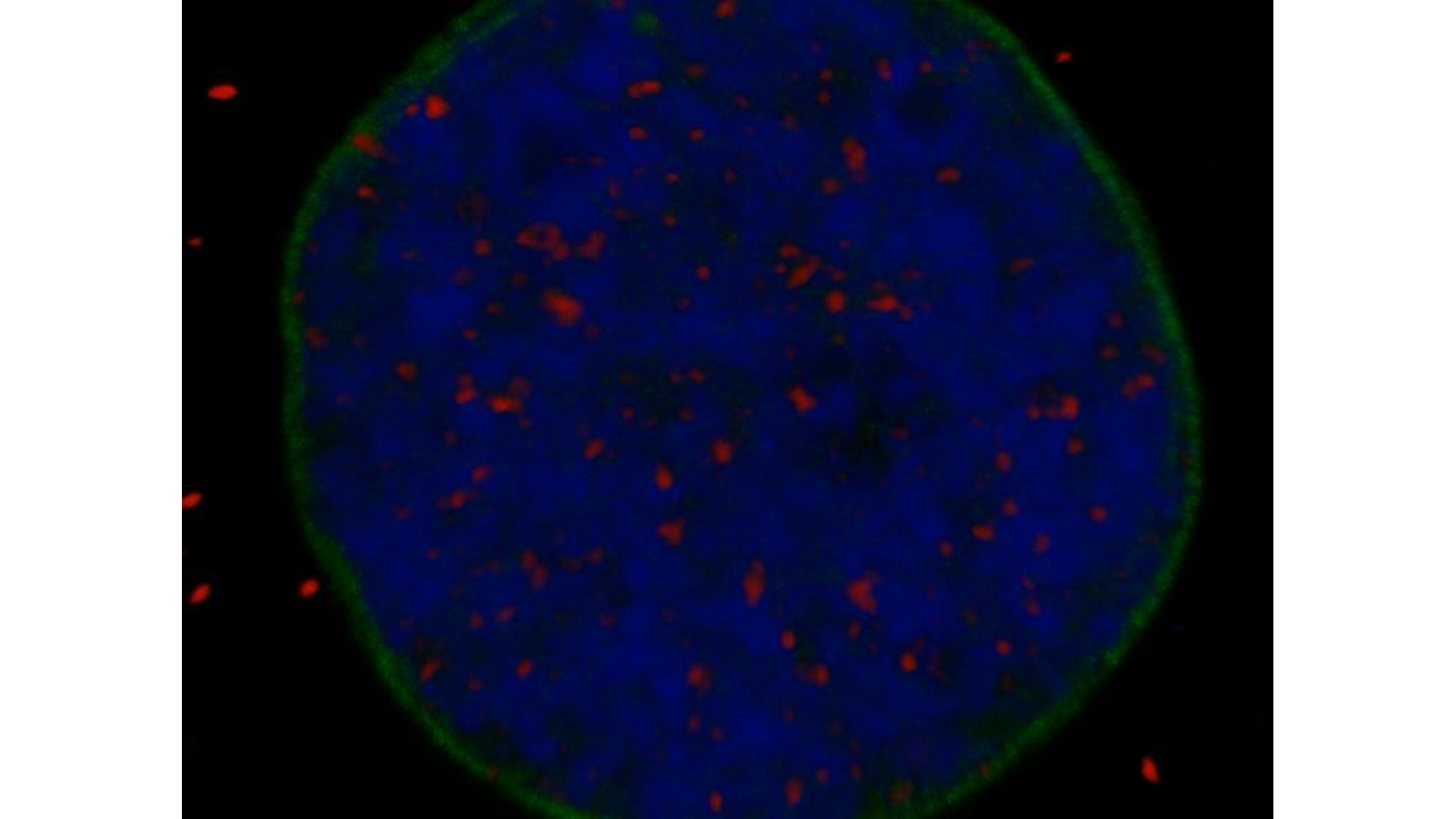

## Slide 2
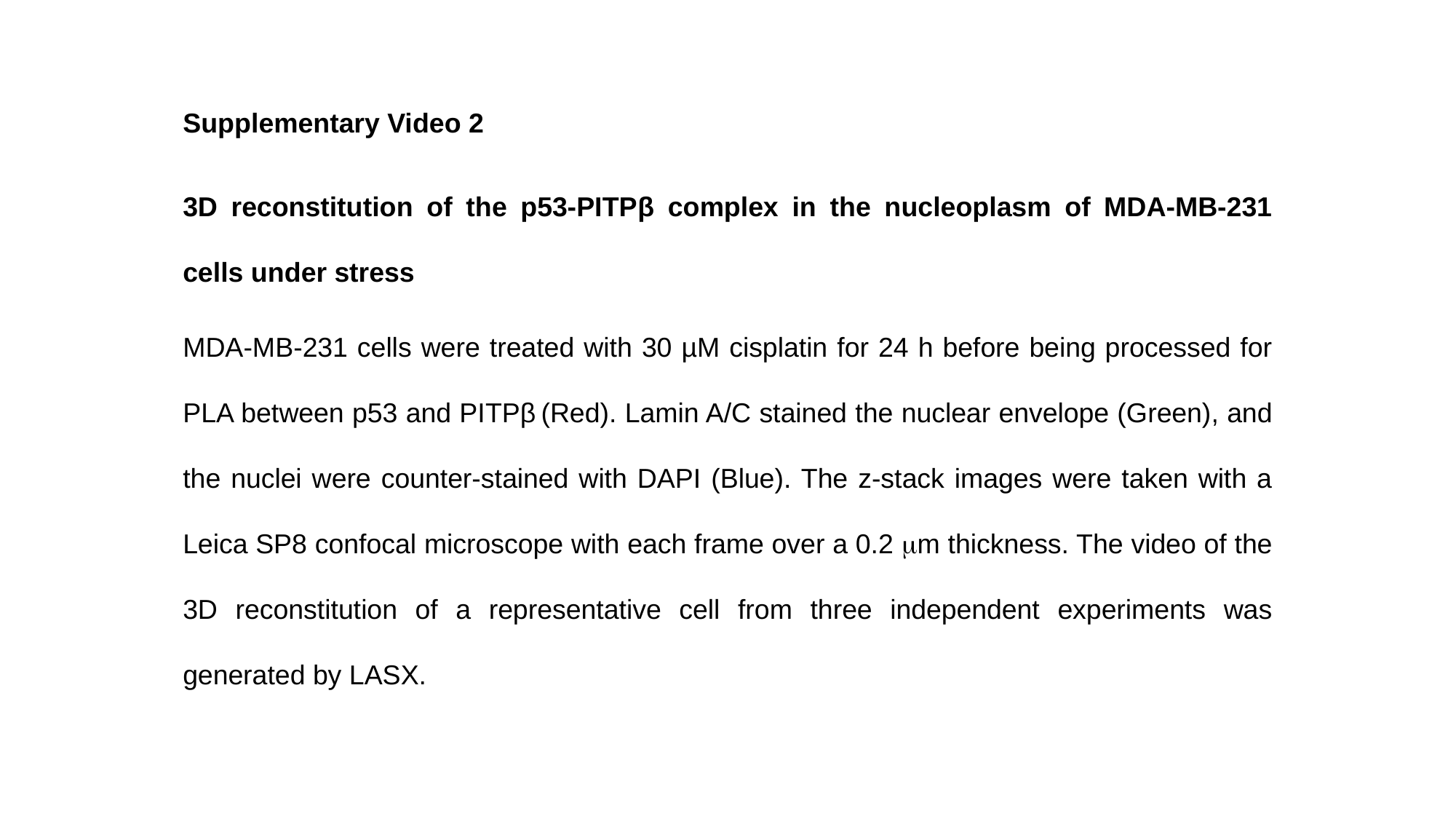

Supplementary Video 2
3D reconstitution of the p53-PITPβ complex in the nucleoplasm of MDA-MB-231 cells under stress
MDA-MB-231 cells were treated with 30 µM cisplatin for 24 h before being processed for PLA between p53 and PITPβ (Red). Lamin A/C stained the nuclear envelope (Green), and the nuclei were counter-stained with DAPI (Blue). The z-stack images were taken with a Leica SP8 confocal microscope with each frame over a 0.2 μm thickness. The video of the 3D reconstitution of a representative cell from three independent experiments was generated by LASX.
