# Supplementary material for: Lipid transfer proteins and PI4KIIα initiate nuclear p53-phosphoinositide signaling
Source: J Biol Chem. 2026 May 8;302(6):113123. doi: 10.1016/j.jbc.2026.113123 (PMC13260207; doi:10.1016/j.jbc.2026.113123)
Supplement: Supplementary Figures [file mmc3.pdf]

Extended Data Fig. 1

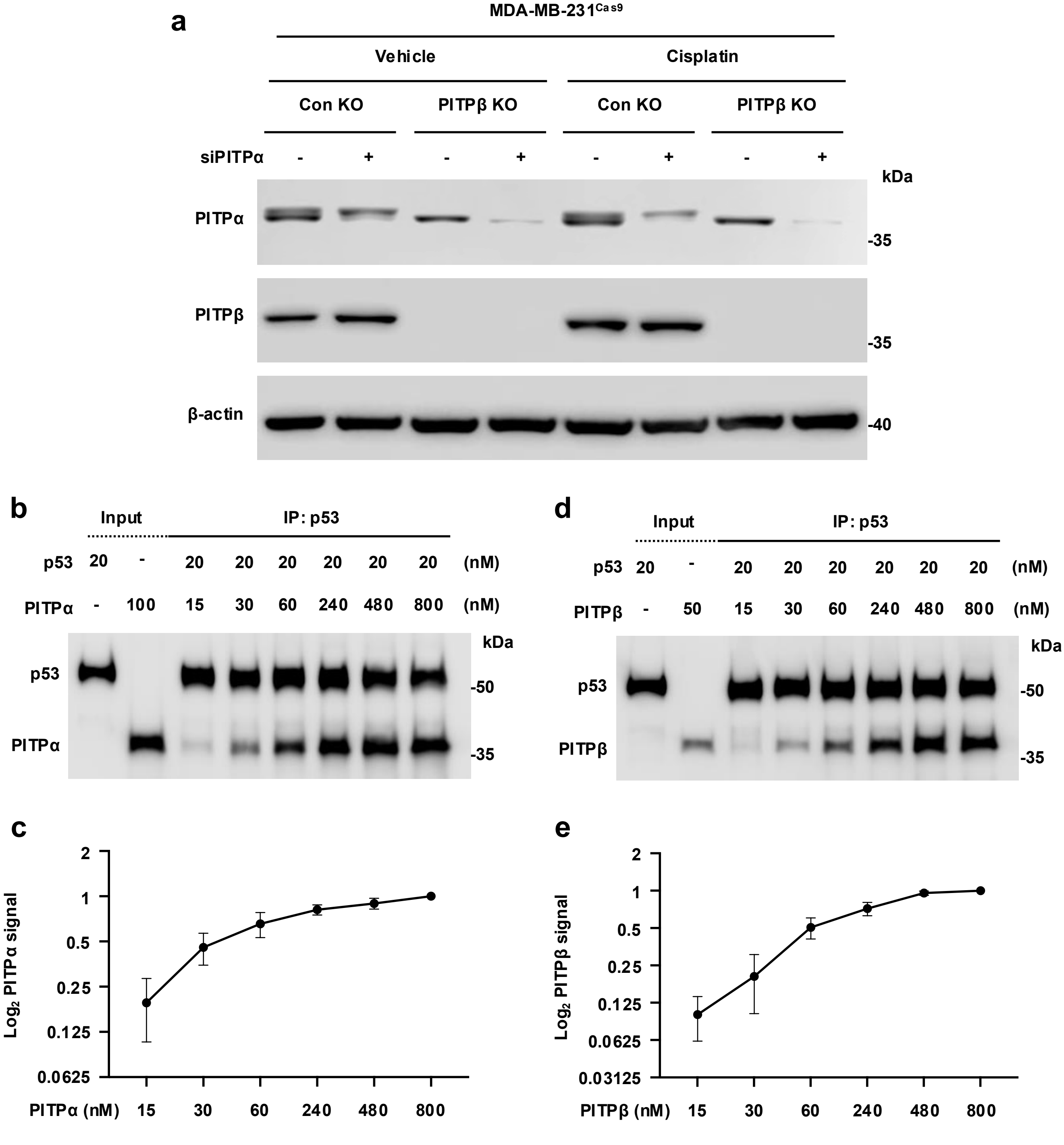

Extended Data Figure 1. PITPα/β validation and interaction with p53

a, MDA-MB-231[Cas9] cells with PITPβ KO and control non-targeted KO were transfected with control siRNAs or siRNAs against PITPα. After 48 h, cells were processed for WB to analyze PITPα and PITPβ expression. n=3 independent experiments.

b-e, *In vitro* binding of recombinant p53 and PITPα (**b-c**) and p53 and PITPβ (**d-e**). Anti-p53 antibody-conjugated agarose was incubated with constant p53 and increasing PITPα or PITPβ protein. p53 was then IPed and analyzed by WB and quantified by ImageJ for p53-bound PITPα (**c**) and p53-bound PITPβ (**e**). n=3 independent experiments.

For all graphs, data are presented as the mean ± SD.

Extended Data Fig. 2

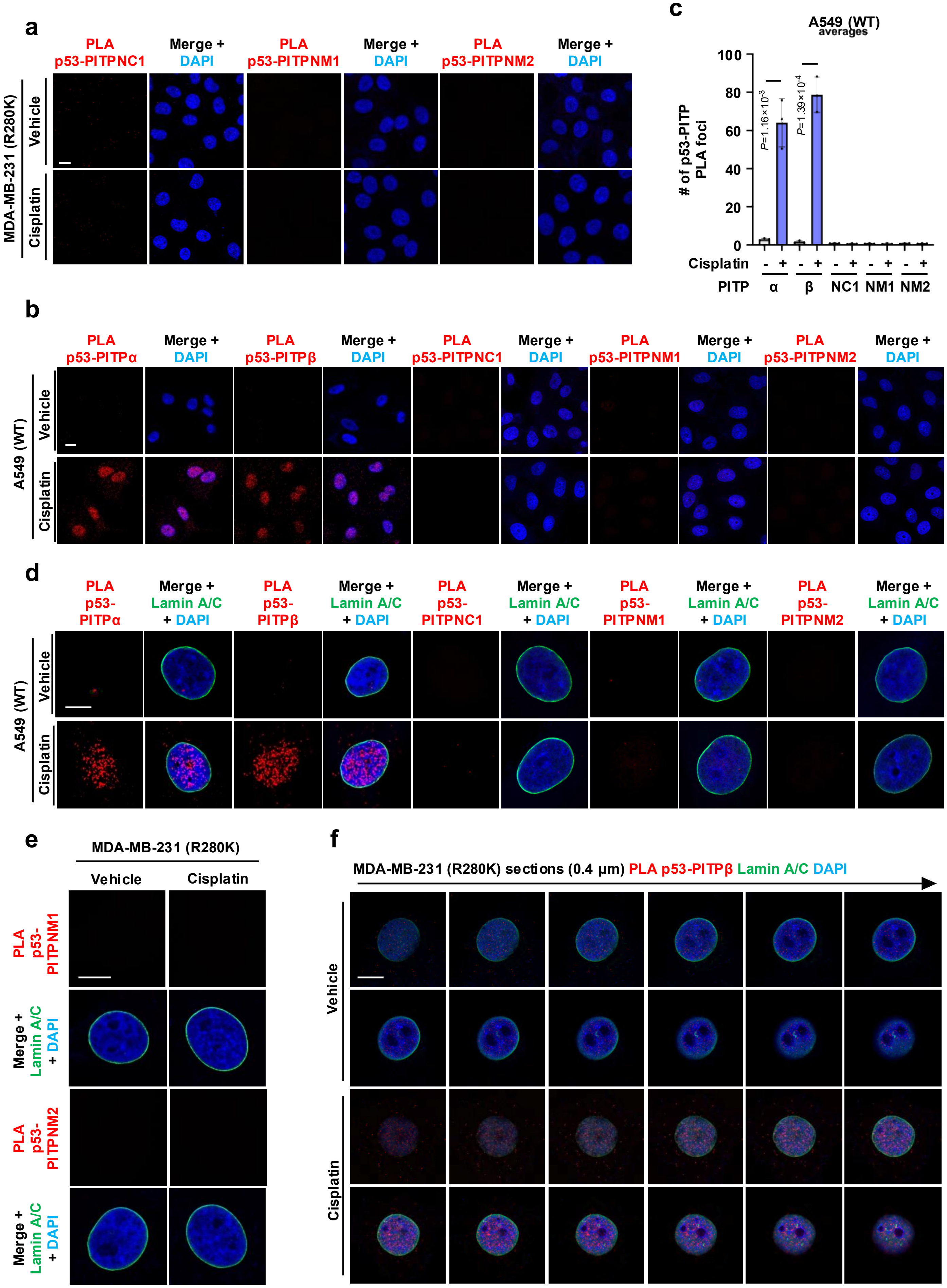

Extended Data Figure 2. p53 interacts with class I PITPs in the nucleus in response to stress

a, PLA of p53-PITPα/PITPβ/PITPNC1/PITPNM1/PITPNM2 in MDA-MB-231 cells treated with vehicle or 30 μM cisplatin for 24 h. n=3, 10 cells from each independent experiment. *p* value denotes two-sided paired t-test. See expanded images in Fig. 1j and quantification in Fig. 1k.

b-c, PLA of p53-PITPα/PITPβ/PITPNC1/PITPNM1/PITPNM2 in A549 cells treated with vehicle or 30 μM cisplatin for 24 h. The nuclear p53-PITP foci were quantified (**c**). n=3, average values were calculated from 10 cells from each independent experiment. *p* value denotes two-sided paired t-test.

d, PLA of p53-PITPα/PITPβ/PITPNC1/PITPNM1/PITPNM2 overlaid with the nuclear envelope marker Lamin A/C in A549 cells treated with vehicle or 30 μM cisplatin for 24 h. The nuclei were counterstained by DAPI. n=3 independent experiments.

e, PLA of p53-PITPα/PITPβ/PITPNC1/PITPNM1/PITPNM2 overlaid with the nuclear envelope marker Lamin A/C in MDA-MB-231 cells treated with vehicle or 30 μM cisplatin for 24 h. The nuclei were counterstained by DAPI. n=3 independent experiments. See expanded images in Fig. 1l.

f, 3D sections of p53-PITPβ PLA foci overlaid with Lamin A/C in MDA-MB-231 treated with vehicle or 30 μM cisplatin for 24 h. The nuclei were counterstained by DAPI. Each frame of the 3D sections was over a 0.2 μm thickness.

For all graphs, data are presented as the mean ± SD. Scale bar, 5 μm.

Extended Data Fig. 3

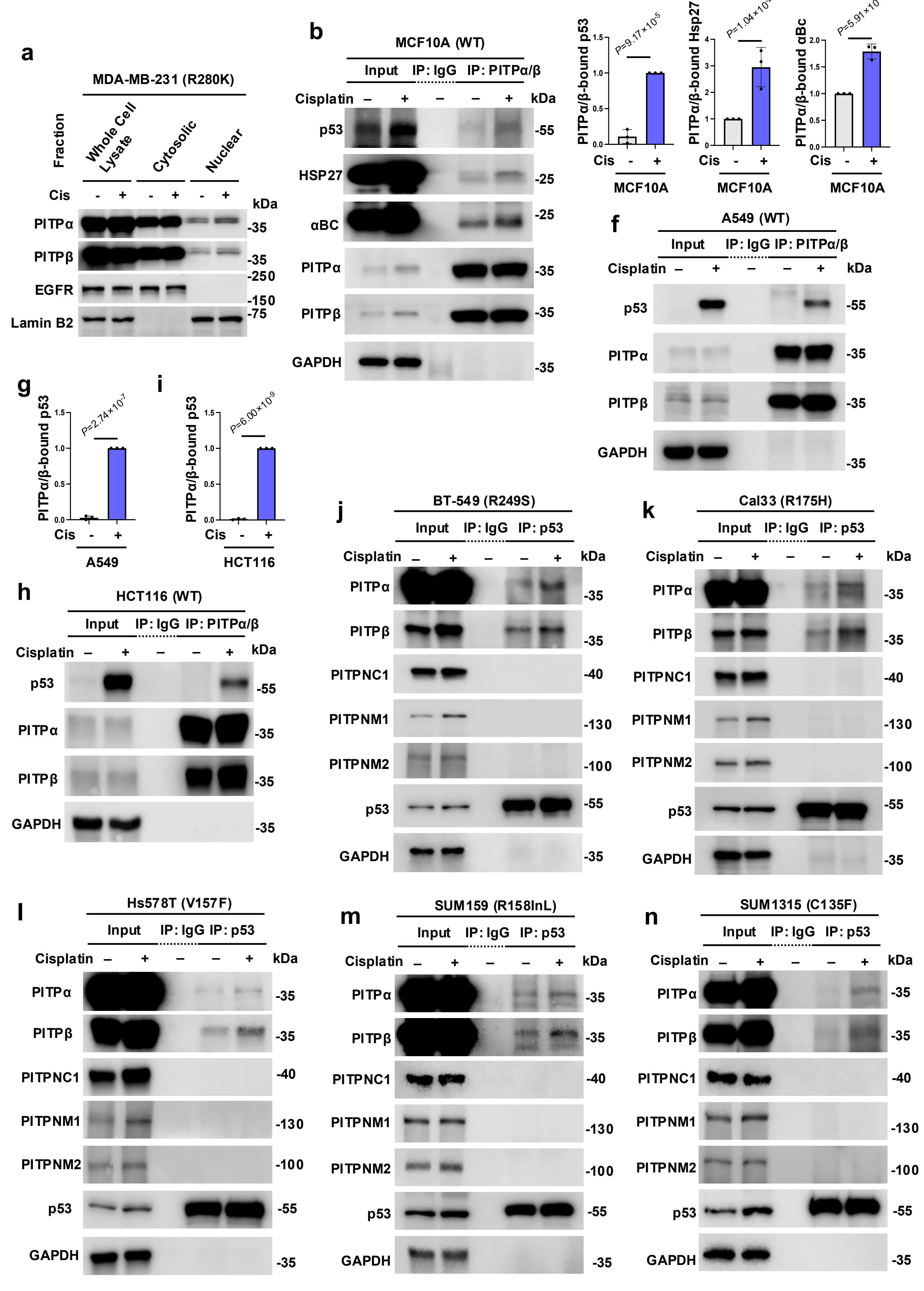

Extended Data Figure 3. p53 interacts with class I PITPs in multiple cell lines

a, MDA-MB-231 cells were treated with vehicle or cisplatin for 24 h. Cells were then fractionated using a detergent-based extraction to produce lysates from cytosolic and nuclear fractions and analyzed via WB. n=3 independent experiments.

b-e, Co-IP of p53/Hsp27/αB-crystallin (αBC) with the shared epitope of PITPα/β in MCF10A cells treated with vehicle or 30 μM cisplatin for 24 h. p53 immunoprecipitated (IPed) by PITPα/β (**c**), Hsp27 IPed by PITPα/β (**d**), and αBC IPed by PITPα/β (**e**) were analyzed by WB. n=3 independent experiments.

f-g, Co-IP of p53 with the shared epitope of PITPα/β in A549 cells treated with vehicle or 30 μM cisplatin for 24 h. p53 IPed by PITPα/β was analyzed by WB (**g**). n=3 independent experiments.

h-i, Co-IP of p53 with the shared epitope of PITPα/β in HCT116 cells treated with vehicle or 30 μM cisplatin for 24 h. p53 IPed by PITPα/β was analyzed by WB (**i**). n=3 independent experiments.

j-n, Co-IP of PITPα/PITPβ with p53 in BT549, Cal33, HS578T, SUM159, SUM1315 cells treated with vehicle or 30 μM cisplatin for 24 h. PITPα/PITPβ IPed by p53 and PITPNC1/PITPNM1/PITPNM2 were analyzed by WB. n=3 independent experiments.

For all panels, data are represented as mean ± SD, and the *p* value denotes a two-sided paired t-test.

Extended Data Fig. 4

a

p53-PITPα

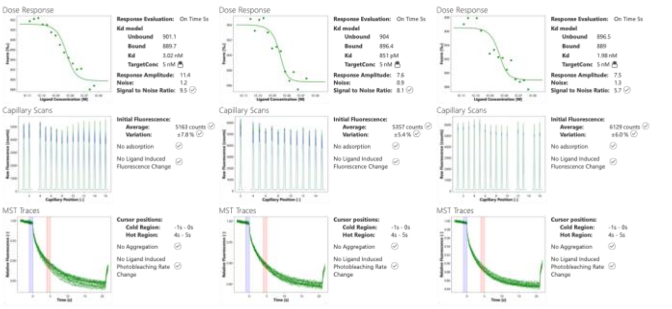

b

p53-PITPα + PI (1μM)

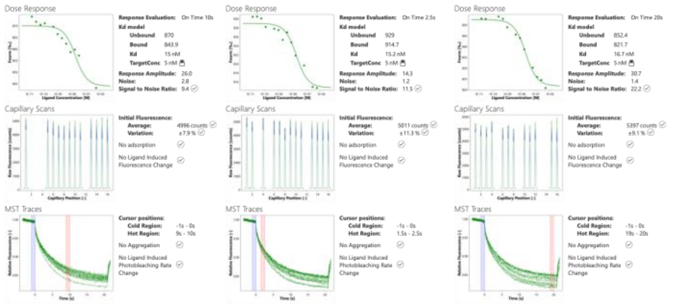

c

p53-PITPβ

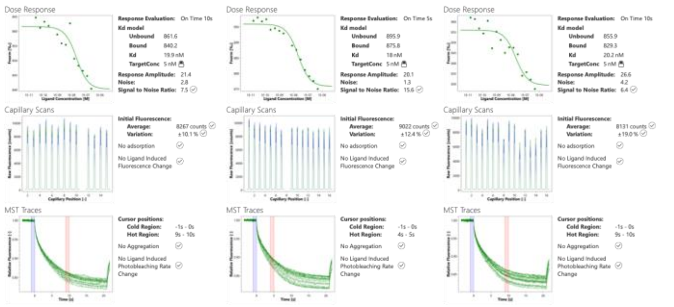

d

p53-PITPβ + PI (1μM)

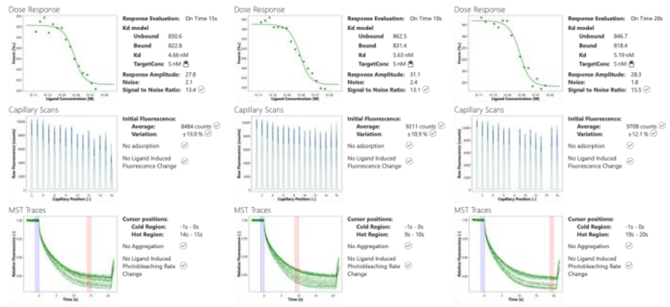

e

p53-PITPNC1

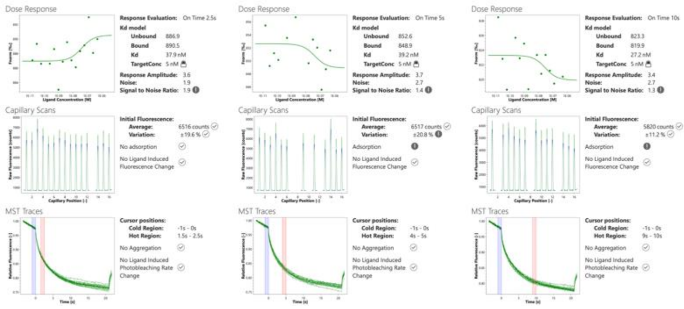

Extended Data Figure 4. MST summary

a-e, The interaction of recombinant fluorescently labelled p53 with PITPα, PITPβ, and PITPNC1 was quantitated by MST assay. A constant concentration of fluorescently labelled p53 (5 nM) was incubated with increasing concentrations of non-labelled ligand with or without the addition of 1 μM PI (micelles) and analyzed using a Monolith NT.115 pico, and the binding affinity was autogenerated by MO. Control v.1.6 software. Triplicate runs are presented for dose response, capillary scans, and MST traces. See average values ± SD in Fig. 1n.

Extended Data Fig. 5

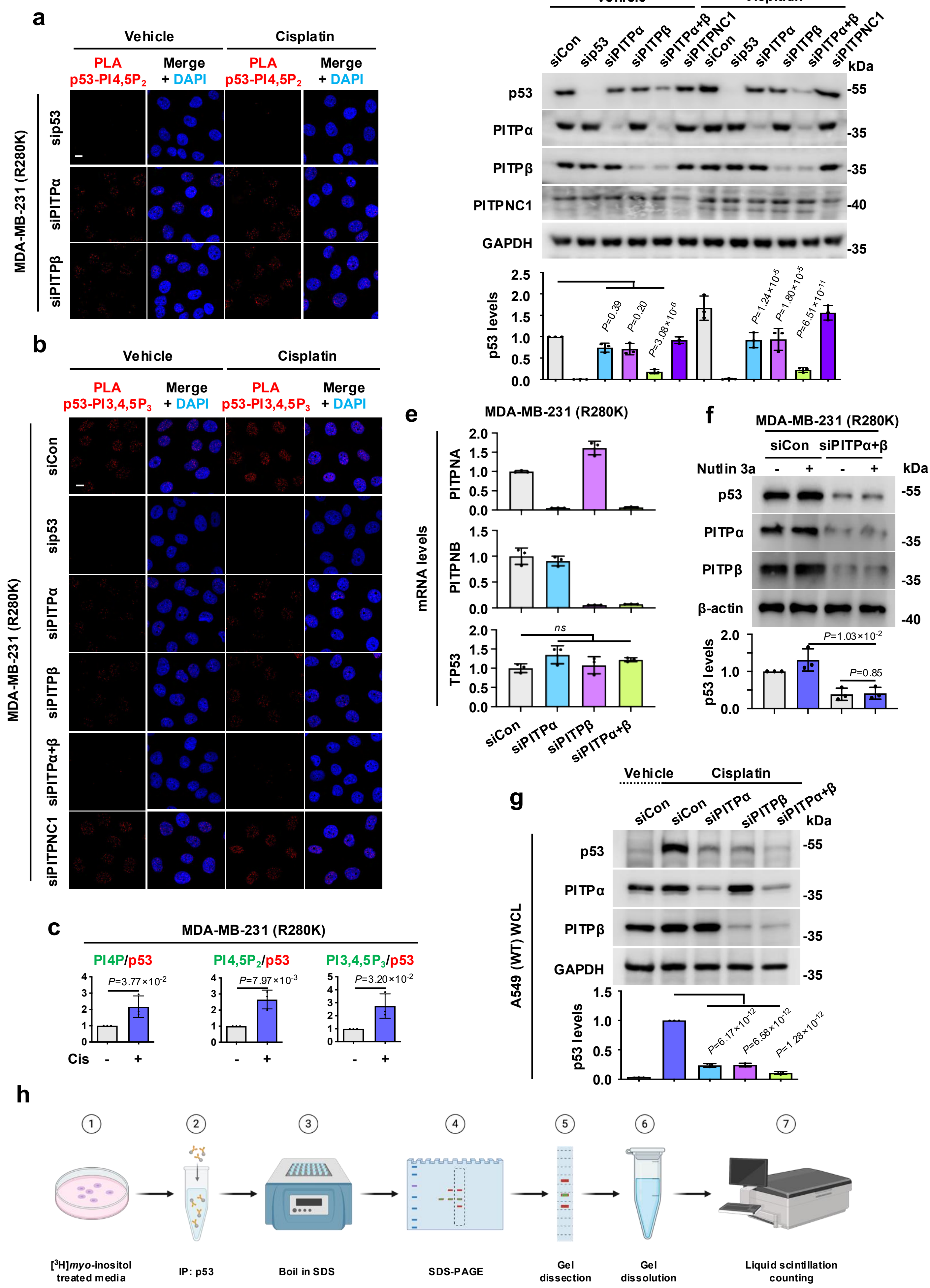

Extended Data Figure 5. PITPα/β regulate p53-PIP_n complexes and p53 levels

a-b, MDA-MB-231 cells were transfected with control siRNAs or siRNAs against p53, PITPα, PITPβ, PITPNC1, or both PITPα and PITPβ. After 24 h, cells were treated with 30 μM cisplatin or vehicle for 24 h before being processed for PLA to detect p53-PI4,$5P_2$ (**a**) and p53-PI3,4,$5P_3$ (**b**) complexes. n=3 independent experiments. See KD validation in Extended Data Fig. 4d, expanded images in Fig. 2a, and quantification in Fig. 2b,c.

c, MDA-MB-231 cells were treated with 30 μM cisplatin or vehicle for 24 h before being processed for IP against p53. p53-bound PI4P/PI4,$5P_2$/PI3,4,$5P_3$ were examined by fluorescent WB in Fig. 2g before being analyzed by imageJ. n=3 independent experiments. *p* value denotes two-sided paired t-test.

d, MDA-MB-231 cells were transfected with control siRNAs or siRNAs against p53, PITPα, PITPβ, PITPNC1, or both PITPα and PITPβ. After 24 h, cells were treated with 30 μM cisplatin or vehicle for 24 h before being processed for WB to validate the KD. The p53 level was quantified by ImageJ. n=3 independent experiments. *p* value denotes ANOVA with Bonferroni's multiple comparisons test.

e, MDA-MB-231 cells transfected with control siRNAs or siRNAs against PITPα, PITPβ, or both PITPα and PITPβ. After 48 h, total RNA was extracted before being processed for qPCR analysis of PITPNA, PITPNB and TP53.

f, MDA-MB-231 cells were transfected with control siRNAs or siRNAs against both PITPα and PITPβ. After 24 h, cells were treated with 10 μM Nutlin 3a or vehicle for 24 h before being processed for WB and analyzed using ImageJ. *p* value denotes two-sided paired t-test. n=3 independent experiments.

g, A549 cells were transfected with control siRNAs or siRNAs against PITPα, PITPβ, or both PITPα and PITPβ. After 24 h, cells were treated with 30 μM cisplatin or vehicle for 24 h before being processed for WB. The p53 level was quantified by ImageJ. n=3 independent experiments. *p* value denotes ANOVA with Bonferroni's multiple comparisons test.

h, A model of the [^{3}H]-inositol lableing workflow used to detect p53-PIP_n complexes. See related data in Fig. 3a-b.

For all panels, data are represented as mean $\pm$ SD. Scale bar, 5 μm.

Extended Data Fig. 6

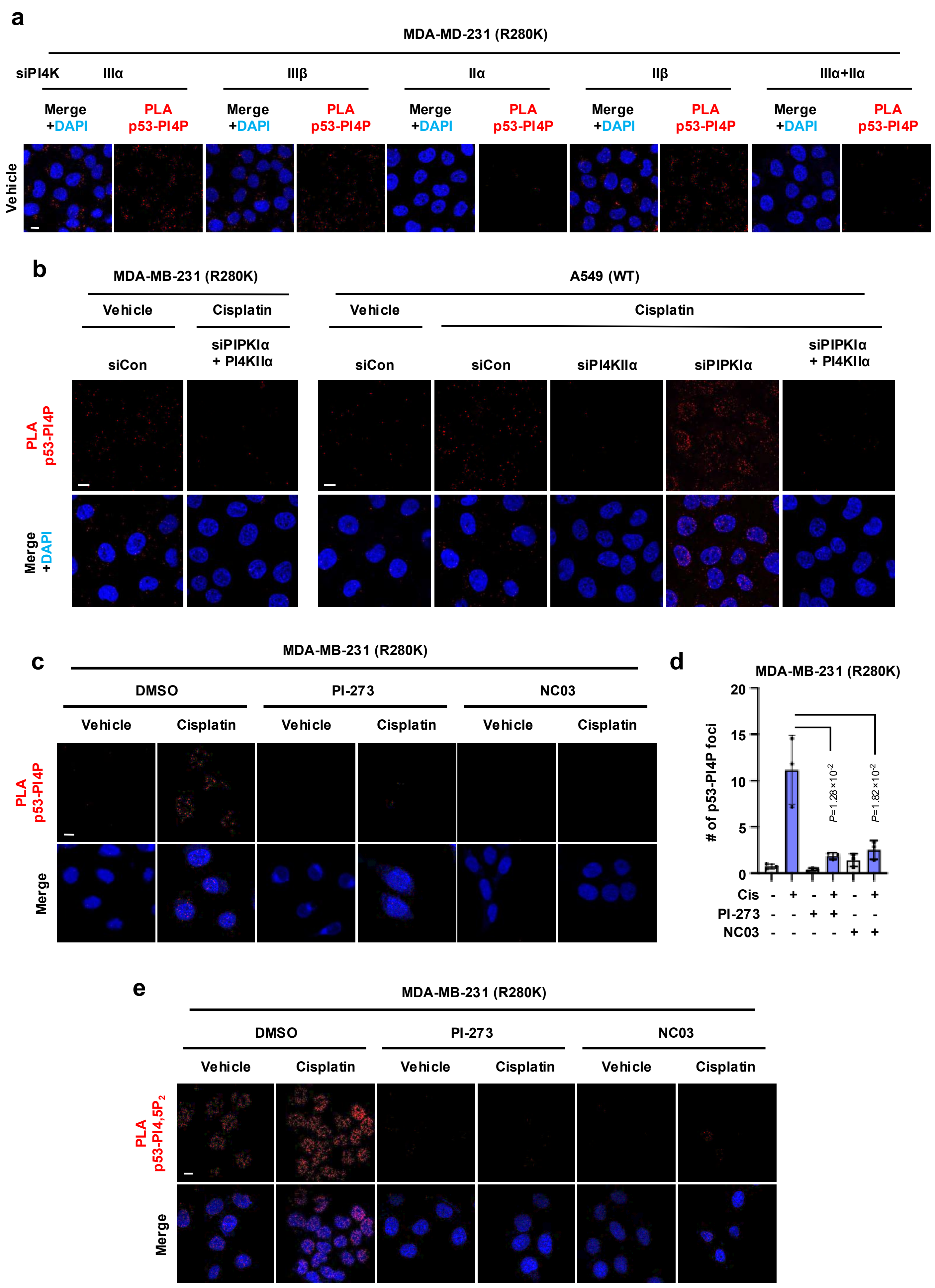

Extended Data Figure 6. PI4KIIα activity is required to generate p53-PI4P

a, MDA-MB-231 cells were transfected with control siRNAs or siRNAs against PI4KIIIα, PI4KIIIβ, PI4KIIα, PI4KIIβ or both PI4KIIα and PI4KIIIα. After 24 h, cells were treated with 30 μM cisplatin or vehicle for 24 h before being processed for PLA to detect p53-PI4P complexes (**b**). n=3 independent experiment. See expanded images in Fig. 4a and quantification in Fig. 4b.

b, MDA-MB-231 and A549 cells were transfected with control siRNAs or siRNAs against PI4KIIα, PIPKIα or both PI4KIIα and PIPKIα. After 24 h, cells were treated with 30 μM cisplatin or vehicle for 24 h before being processed for PLA to detect p53-PI4P complexes. n=3 independent experiment. See KD confirmation in Fig. 4d, expanded images in Fig. 4e, and quantification in Fig. 4f.

c-d, MDA-MB-231 cells were treated with vehicle or cisplatin in combination with DMSO as a control, PI-273, or NC03 for 24 h before being processed for PLA to detect p53-PI4P complexes and analyzed using ImageJ (**d**). n=3, average values were calculated from 15 cells from each independent experiment. *p* value denotes two-sided paired t-test.

e, MDA-MB-231 cells were treated with vehicle or cisplatin in combination with DMSO as a control, PI-273, or NC03 for 24 h before being processed for PLA to detect p53-PI4,5P_2 complexes. n=3 independent experiments. See quantification in Fig. 4g.

For all panels, scale bar, 5 μm.

Extended Data Fig. 7

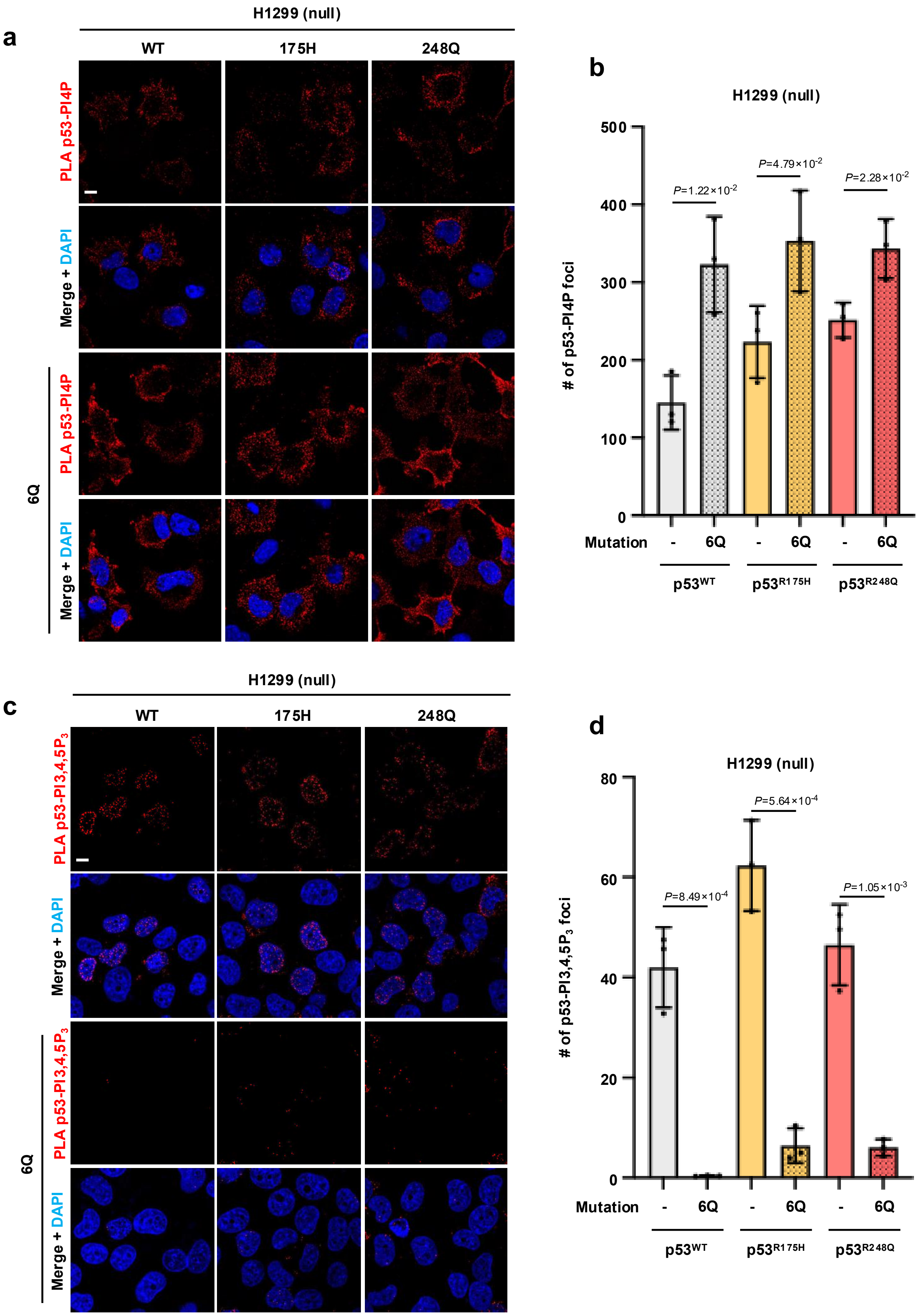

Extended Data Figure 7. p53^{6Q} mutants still form p53-PI4P complexes

a-b, H1299 cells transfected with constructs expressing p53 WT, WT6Q, 175H, 175H6Q, 248Q and 248Q6Q were treated with 30 µM cisplatin for 24 h. Then, the cells were processed for PLA to detect p53-PI4P complexes (**b**). Nuclei were counterstained by DAPI. n=3, average values were calculated from 10 cells from each independent experiment. *p* value denotes two-sided paired t-test.

c-d, H1299 cells transfected with constructs expressing p53 WT, WT6Q, 175H, 175H6Q, 248Q, and 248Q6Q were treated with 30 µM cisplatin for 24 h. Then, the cells were processed for PLA to detect p53-PI3,4,5P_3 complexes (**d**). Nuclei were counterstained by DAPI. n=3, average values were calculated from 10 cells from each independent experiment. *p* value denotes two-sided paired t-test.

For all panels, data are represented as mean ± SD. Scale bar, 5 µm.

Extended Data Fig. 8

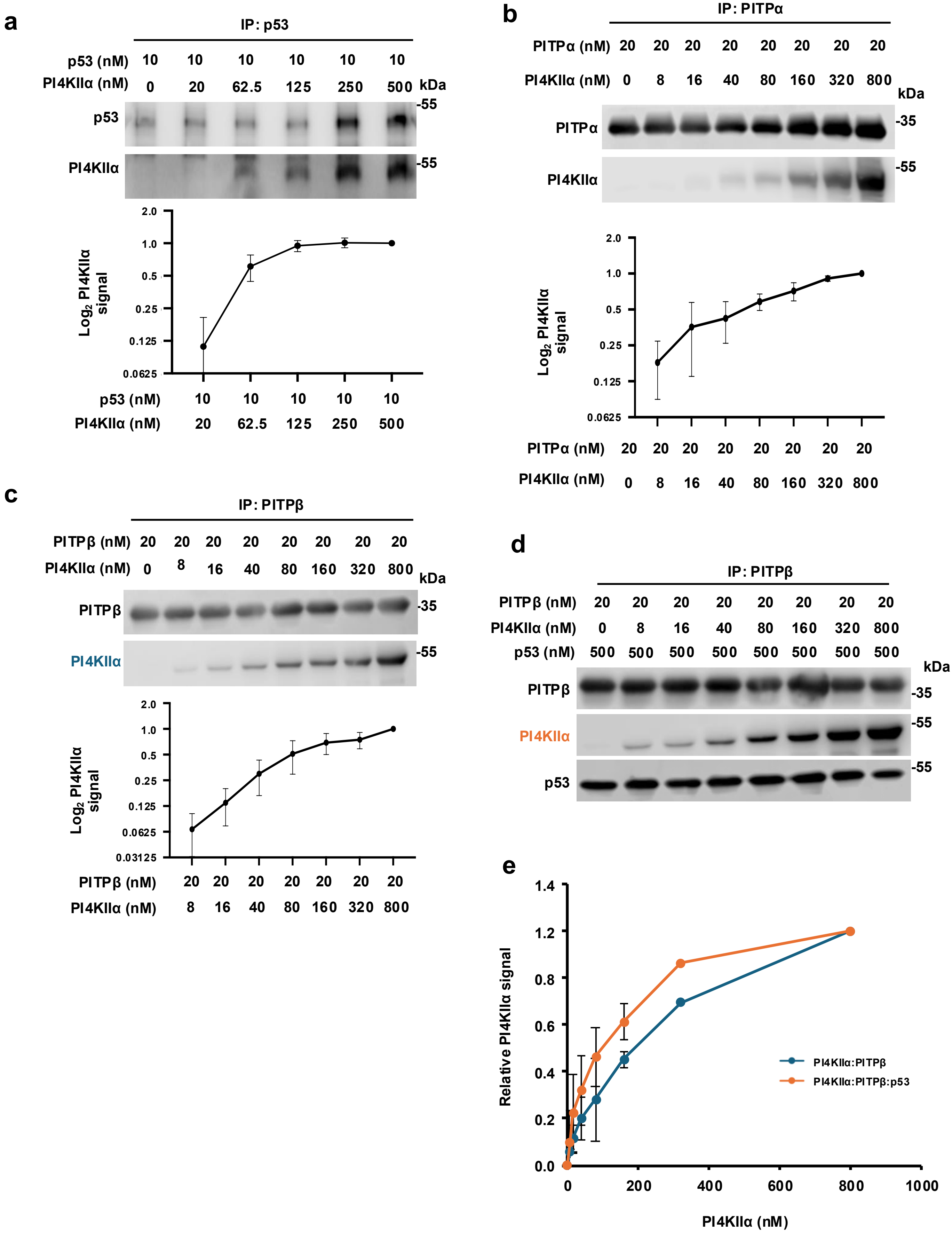

Extended Data Figure 8. PI4KIIα interacts with PITPα/β and p53 cooperatively

a-c, *In vitro* binding of recombinant PI4KIIα and p53 (**a**), PI4KIIα and PITPα (**b**), and PI4KIIα and PITPβ (**c**). p53, PITPα, or PITPβ immobilized on the indicated antibody-conjugated agarose was incubated with PI4KIIα protein. p53, PITPα or PITPβ was IPed and p53-bound PI4KIIα (**a**), PITPα-bound PI4KIIα (**b**) and PITPβ-bound PI4KIIα (**c**) was analyzed by WB and quantified by ImageJ. n=3 independent experiments.

d-e, *In vitro* binding of recombinant PITPβ and PI4KIIα with and without p53. Anti-PITPβ antibody-conjugated agarose was incubated with constant PITPβ and increasing PI4KIIα protein in the presence (**e**) of p53 (500 nM). PITPβ was then IPed, analyzed by WB, quantified by ImageJ for PITPβ-bound PI4KIIα, and compared to PITPβ and PI4KIIα binding without p53 (**c**)(**f**). n=3 independent experiments.

For all panels, data are presented as the mean ± SD.

Extended Data Fig. 9

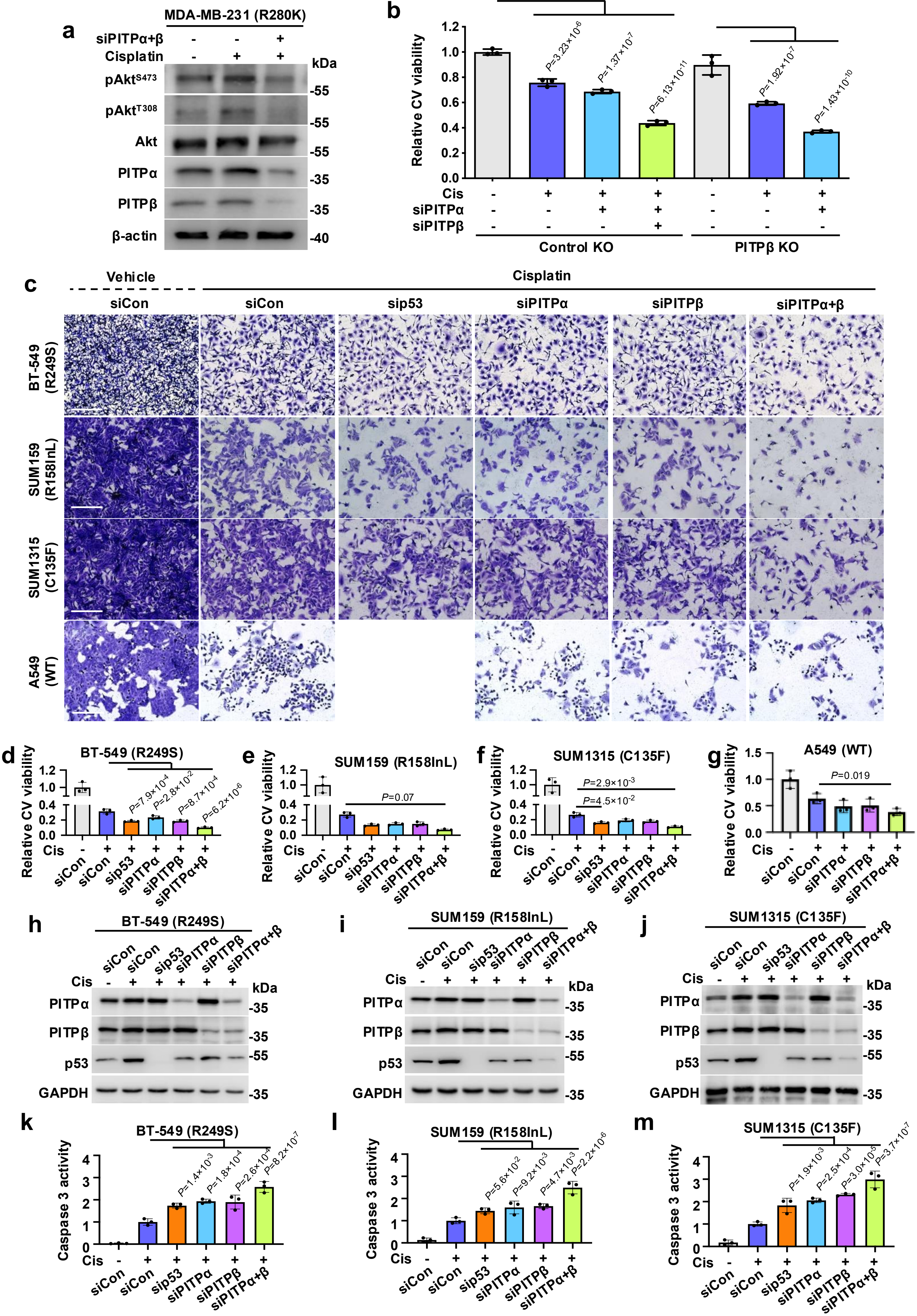

Extended Data Figure 9. PITPα/β regulate Akt activation and chemoresistance

a, MDA-MB-231 cells were transfected with control siRNAs or siRNAs against both PITPα and PITPβ. After 24 h, cells were treated with 30 μM cisplatin or vehicle for 24 h before being processed for WB. n=3 independent experiments.

b, MDA-MB-231^{Cas9} cells with PITPβ KO and control non-targeted KO were transfected with control siRNAs or siRNAs against PITPα, PITPβ, or both PITPα and PITPβ. After 24 h, cells were treated with 30 μM cisplatin or vehicle for 24 h before being processed for Crystal Violet viability assay. Viability was quantified based on the extracted dye using a plate reader. *p* value denotes ANOVA with Bonferroni's multiple comparisons test. n=3 independent experiments. See representative images in Fig. 5h.

c-j, BT-549, SUM159, and SUM1315 cells were transfected with control siRNAs or siRNAs against p53, PITPα, PITPβ, or both PITPα and PITPβ. A549 cells were transfected with control siRNAs or siRNAs against PITPα, PITPβ, or both PITPα and PITPβ. After 24 h, cells were treated with 30 μM cisplatin or vehicle for 24 h before being processed for Crystal Violet viability assay. The cells were imaged by an EVOS M5000 microscope (**c**) and quantified based on the extracted dye using a plate reader (**d-f**). The KD was validated by WB (**g-i**). *p* value denotes ANOVA with Bonferroni's multiple comparisons test. n=3 independent experiments.

k-m, BT-549, SUM159, and SUM1315 cells were transfected with control siRNAs or siRNAs against p53, PITPα, PITPβ, or both PITPα and PITPβ. After 24 h, cells were treated with 30 μM cisplatin or vehicle for 24 h before being processed for a caspase 3 activity assay. *p* value denotes ANOVA with Bonferroni's multiple comparisons test. n=3 independent experiments.

For all graphs, data are presented as the mean ± SD. Scale bar, 300 μm.

Extended Data Fig. 10

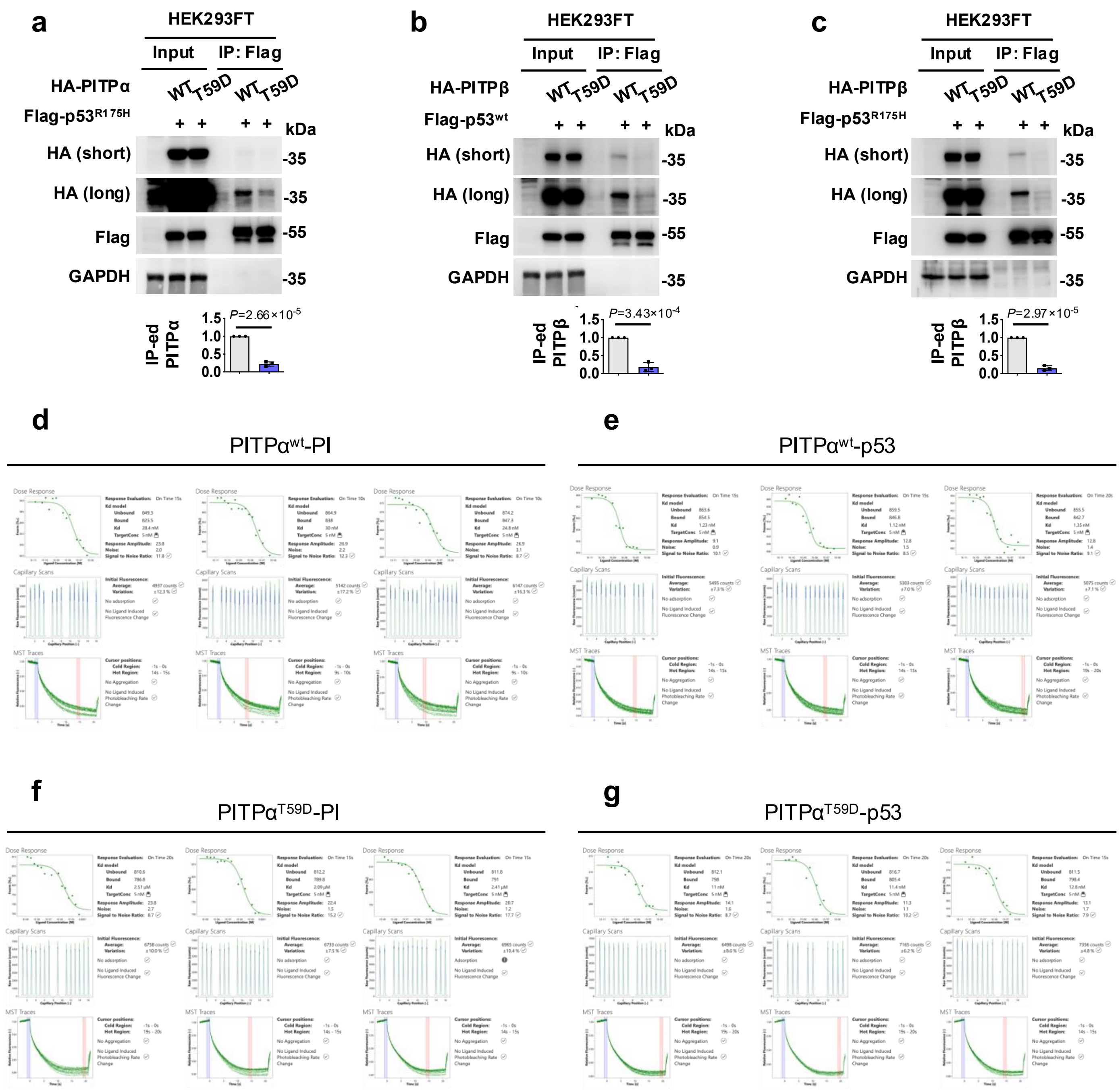

Extended Data Figure 10. PITPs require PI binding to promote oncogenicity

a, HEK293FT cells were co-transfected with either HA-tagged wild-type PITPα or PI binding-defective mutant T59D PITPα together with Flag-tagged mutant $p53^{R175H}$. After 48 h, cells were processed for IP against Flag-tag and analyzed by WB. *p* value denotes two-sided paired t-test. n=3 independent experiments.

b, HEK293FT cells were co-transfected with either HA-tagged wild-type PITPβ or PI binding-defective mutant T59D PITPβ together with Flag-tagged $p53^{wt}$. After 48 h, cells were processed for IP against Flag-tag and analyzed by WB. *p* value denotes two-sided paired t-test. n=3 independent experiments.

c, HEK293FT cells were co-transfected with either HA-tagged wild-type PITPβ or PI binding-defective mutant T59D PITPβ together with Flag-tagged mutant $p53^{R175H}$. After 48 h, cells were processed for IP against Flag-tag and analyzed by WB. *p* value denotes two-sided paired t-test. n=3 independent experiments.

d-g, The interaction of recombinant fluorescently labelled $PITP\alpha^{wt}$ and $PITP\alpha^{T59D}$ with p53 and PI was quantitated by MST assay. A constant concentration of fluorescently labelled PITPα (5 nM) was incubated with increasing concentrations of non-labelled ligand and analyzed using a Monolith NT.115 pico, and the binding affinity was autogenerated by MO. Control v.1.6 software. Triplicate runs are presented for dose response, capillary scans, and MST traces. See average values ± SD in Fig. 6b.

For all graphs, data are presented as the mean ± SD.

Extended Data Fig. 11

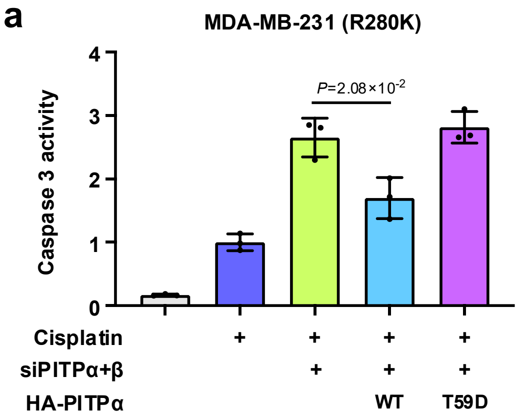

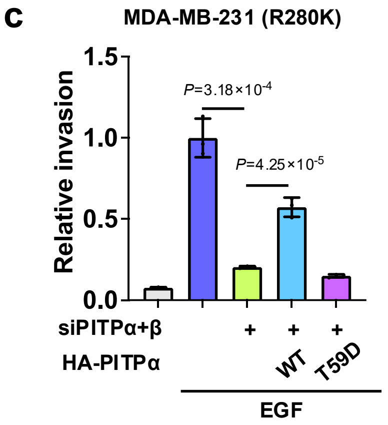

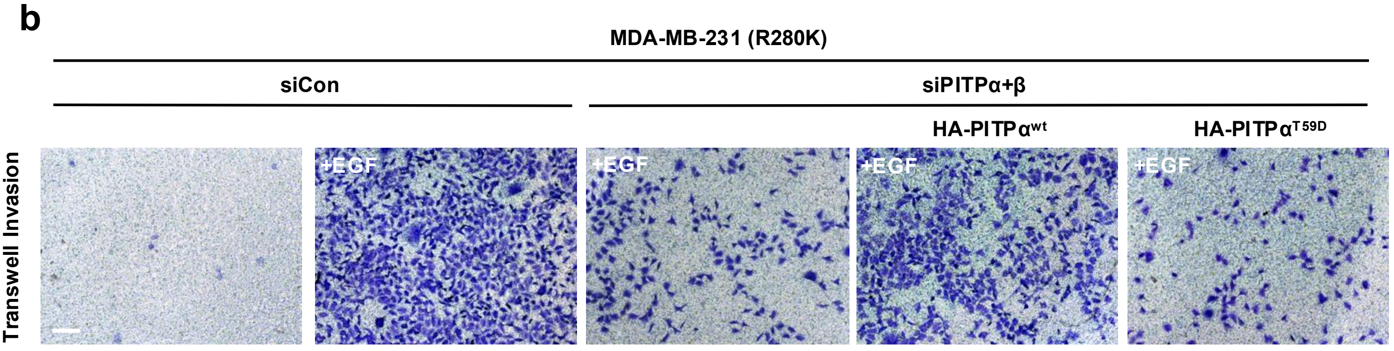

Extended Data Figure 11. PITPs require PI binding to function with p53

a, MDA-MB-231 cells were transfected with control siRNAs or siRNAs against the 3'UTR of both PITPα and PITPβ for 24 h. The cells were then transfected with either HA-tagged wild-type PITPα or PI binding-defective mutant T59D PITPα. After 24 h, cells were treated with 30 μM cisplatin or vehicle for 24 h before being processed for a caspase 3 activity assay. p value denotes two-sided paired t-test.

b-c, MDA-MB-231 cells were transfected with control siRNAs or siRNAs against the 3'UTR of both PITPα and PITPβ for 24 h. The cells were then transfected with either HA-tagged wild-type PITPα or PI binding-defective mutant T59D PITPα. After 24 h, cells were serum starved for an additional 24 h and then scored for invasion through Laminin-coated transwell inserts with 8 μm pores using 10 ng/ml EGF as a chemoattractant for 16 h. The invading cells at the insert bottom were stained with Crystal Violet, imaged (**b**), and quantified (**c**) based on the extracted dye using a plate reader. n=3 independent experiments. *p* value denotes two-sided paired t-test. Scale bar, 300 μm.

For all graphs, data are presented as the mean ± SD.
